# The Sebaceous Gland: A Key Player in the Balance Between Homeostasis and Inflammatory Skin Diseases

**DOI:** 10.3390/cells14100747

**Published:** 2025-05-20

**Authors:** Sarah Mosca, Monica Ottaviani, Stefania Briganti, Anna Di Nardo, Enrica Flori

**Affiliations:** Laboratory of Cutaneous Physiopathology and Integrated Center of Metabolomics Research, San Gallicano Dermatological Institute, IRCCS, 00144 Rome, Italy; sarah.mosca@ifo.it (S.M.); anna.dinardo@ifo.it (A.D.N.); enrica.flori@ifo.it (E.F.)

**Keywords:** sebaceous gland, sebocyte, inflammatory skin disorders, acne, hidradenitis suppurativa, rosacea, seborrheic dermatitis, atopic dermatitis, psoriasis, eosinophilic pustular folliculitis, melasma

## Abstract

The sebaceous gland (SG) is an integral part of the pilosebaceous unit and is a very active and dynamic organ that contributes significantly to the maintenance of skin homeostasis. In addition to its primary role in sebum production, the SG is involved in the maintenance of skin barrier function, local endocrine/neuroendocrine function, the innate immune response, and the regulation of skin bacterial colonization. Structural and functional alterations of SGs leading to the dysregulation of sebum production/composition and immune response may contribute to the pathogenesis of inflammatory dermatoses. This review summarises the current knowledge on the contribution of SGs to the pathogenesis of common inflammatory skin diseases. These findings are crucial for the development of more effective therapeutic strategies for the treatment of inflammatory dermatoses.

## 1. Introduction

Sebaceous glands (SGs) are an integral component of the pilosebaceous unit, playing a critical role in maintaining skin homeostasis and barrier function. Their distribution across the human body varies significantly, with the highest density found in seborrheic areas such as the face, scalp, chest, and upper back, while the palms and soles are entirely devoid of them [1]; the scalp and forehead exhibit particularly high SG density, ranging from 400 to 900 glands per cm^2^ [2].

SGs originate from the same stem cell population as hair follicles and the epidermis, emerging during embryogenesis as outgrowths of the hair follicles [3,4]. This SG-forming bulge is located beneath the follicle’s infundibulum and superior to the hair follicle stem cell niche [5].

Historically, SGs were believed to function primarily in sebum production, with early research focusing more on other skin structures such as the epidermis and hair follicles [6]. However, recent advances in sebum research have revealed the critical role of SGs in acne pathogenesis, prompting extensive studies on this topic [7,8,9,10]. Sebum, once considered a passive byproduct, is now recognised as a complex lipid mixture essential for skin lubrication, barrier protection, pH regulation, and antimicrobial defence [11,12,13,14]. SGs have gained recognition over the past few decades as dynamic endocrine organs that respond to hormonal (e.g., androgens) and neuroendocrine (e.g., corticotropin-releasing hormone) stimuli, modulate immune responses via antimicrobial peptides and cytokines, and regulate cutaneous inflammation. These numerous functions have led several scientists to describe the SG as “the brain of the skin” [15], considering its involvement in both physiological and pathological skin conditions.

This review summarises the current knowledge of SGs and sebocytes in skin pathophysiology, particularly their involvement in common skin disorders mediated by chronic inflammation and microbiome dysregulation.

## 2. Materials and Methods

We performed a comprehensive PubMed literature search for relevant studies published between 2000 and 2025, including human and animal studies. Other articles were identified by checking the reference lists of all articles. Searches were conducted for all English peer-reviewed articles, with a preference for more recent literature. Our search was performed from 27 January 2025 to 9 April 2025. The search strategy employed the following terms: “sebaceous glands [MeSH]” OR “sebaceous gland [keyword]” OR “sebocytes [keyword]”; “inflammatory skin disorders [keyword]” AND “sebaceous glands [MeSH]” OR “sebaceous gland [keyword]” OR “sebocytes [keyword]”; “acne vulgaris [MeSH]” OR “Acne [keyword]”; “acne vulgaris [MeSH]” OR “Acne [keyword]” AND “sebaceous glands [MeSH]” OR “sebaceous gland [keyword]” OR “sebocytes [keyword]”; “seborrheic dermatitis [MeSH]” OR “seborrheic dermatitis [keyword]”; “seborrheic dermatitis [MeSH]” OR “seborrheic dermatitis [keyword]” AND “sebaceous glands [MeSH]” OR “sebaceous gland [keyword]” OR “sebocytes [keyword]”; “eosinophilic pustular folliculitis [MeSH]” OR “eosinophilic pustular folliculitis [keyword]”; “eosinophilic pustular folliculitis [MeSH]” OR “eosinophilic pustular folliculitis [keyword]” AND “sebaceous glands [MeSH]” OR “sebaceous gland [keyword]” OR “sebocytes [keyword]”; “rosacea [MeSH]” OR “rosacea [keyword]”; “rosacea [MeSH]” OR “rosacea [keyword]” AND “sebaceous glands [MeSH]” OR “sebaceous gland [keyword]” OR “sebocytes [keyword]”; “atopic dermatitis [MeSH]” OR “atopic dermatitis [keyword]”; “atopic dermatitis [MeSH]” OR “atopic dermatitis [keyword]” AND “sebaceous glands [MeSH]” OR “sebaceous gland [keyword]” OR “sebocytes [keyword]”; “psoriasis [MeSH]” OR “psoriasis [keyword]”; “psoriasis [MeSH]” OR “psoriasis [keyword]” AND “sebaceous glands [MeSH]” OR “sebaceous gland [keyword]” OR “sebocytes [keyword]”; “hidradenitis suppurativa [MeSH]” OR “hidradenitis suppurativa [keyword]”; “ hidradenitis suppurativa [MeSH]” OR “hidradenitis suppurativa [keyword]” AND “sebaceous glands [MeSH]” OR “sebaceous gland [keyword]” OR “sebocytes [keyword]”; “melasma [MeSH]” OR “melasma [keyword]”; “melasma [MeSH]” OR “melasma [keyword]” AND “sebaceous glands [MeSH]” OR “sebaceous gland [keyword]” OR “sebocytes [keyword]”.

## 3. SG Physiology

### 3.1. SG Anatomy and Differentiation

SGs are multi-lobed holocrine glands located in the dermis and are typically associated with hair follicles, forming the pilosebaceous unit [1,16]. An exception is represented by some hair follicle-independent SGs, such as the Meibomian glands on the eyelids, which secrete meibum [17,18], and the SGs of the oral mucosa, also known as Fordyce granules [19,20].

Anatomically, each gland consists of multiple acini, or lobules, composed of sebocytes, which are specialised epithelial cells that undergo a unique differentiation process. These lobules are connected to a central duct that empties into the hair follicle, allowing secretions to reach the skin surface [1,4]. Histologically, SG can be divided into three distinct zones based on the differentiation and activity of sebocytes: the peripheral zone, the middle zone, and the necrotic zone. The outermost layer consists of undifferentiated, proliferating basal cells that serve as the stem cell reservoir for the gland. As sebocytes migrate inward from the peripheral zone, they enter the middle zone, where they begin to differentiate and accumulate lipid droplets. Finally, the innermost zone consists of fully mature sebocytes that have completed their lipid synthesis and accumulation. These cells undergo programmed cell death, during which they disintegrate and release their lipid content as sebum into the glandular duct. The whole process of differentiation of a human sebocyte takes about 7–14 days and is comparable to that of a murine sebocyte [21,22,23,24].

Recent studies have sought to better understand the sebocyte differentiation program and define new therapeutic targets for skin diseases using novel analytical techniques. Schmidt et al. were the first to provide a high-resolution spatial portrait of the SG transcriptional landscape [25]. They discovered that the gradual and continuous differentiation of sebocytes, from the peripheral layer (SEB-B mitotically active sebocytes) to the gland centre (SEB-3 most differentiated sebocytes), reflects the sequential modulation of cellular activities from cell proliferation/sebum production to lysis/apoptosis-related functions. Each differentiation stage is associated with a specific transcriptional profile, which may provide a starting point for identifying new potential candidates for modulating SG function in skin diseases. In addition, it was determined that sebocytes interact with each other and the surrounding extracellular matrix in distinct ways, depending on their differentiation stage, and that structural and functional alterations of these adhesion processes may take place in pathological conditions [26].

### 3.2. Sebum Composition and Regulation

Sebum is an oily, lipid-rich substance composed of triglycerides, wax esters, squalene, cholesterol, and free fatty acids (FFAs) [27,28]. Notably, squalene and wax esters are the most characteristic products of the sebaceous glands and are not found in any other tissues or structures in the body [11,12].

Sebum secretion is primarily regulated by hormones, with androgens, particularly dihydrotestosterone (DHT), playing a crucial role in regulating sebaceous gland activity, stimulating sebocyte proliferation and sebum production [29]. Other hormones, such as oestrogens, glucocorticoids, growth hormone, or the insulin-like growth factor-1 (IGF-1), also modulate sebaceous gland function, highlighting the gland’s responsiveness to systemic physiological changes [11,30]. Sebum production is low in infancy, increases during puberty due to rising androgen levels, and decreases with age [22,31]. This age-dependent variation highlights the role of the gland in skin maturation and ageing. Furthermore, in women, sebum levels appear to increase during the luteal phase of the menstrual cycle [22,32].

Sebum has multiple physiological functions, including skin lubrication, pH regulation, and barrier protection [11,12,33,34]. Data in the literature also demonstrate its role in the defence against environmental and pro-aging insults by acting as an antioxidant and antibacterial barrier [1,23]. Additionally, Banyś et al. demonstrate the role of sebum levels in the directional reflectance of the skin, an important aspect to consider when studying the effects of solar radiation exposure [35]. Sebocytes express several receptors, such as the corticotrophin–releasing hormone (CRH) receptor, melanocortin receptors (MC1-5R), and peroxisome proliferator-activated receptors (PPARs), through which they are modulated by various factors to control sebum synthesis [1,36,37,38].

### 3.3. SG Role in Skin Immunobiology

Data in the literature highlight the crucial role of sebum in maintaining immunological skin homeostasis. Béke et al. explored the immunological differences between sebaceous gland-rich (SGR) and sebaceous gland-poor (SGP) regions of human skin. The research challenges the idea that healthy skin has a unified immunological barrier. It reveals that SGR skin exhibits a distinct innate and adaptive immune environment, characterised by increased chemokine and antimicrobial peptide expression, altered barrier functions, and non-inflammatory T helper (Th17)/IL-17 dominance, compared to SGP skin. Specifically, the study suggests that SGs contribute to unique, non-inflammatory immune surveillance in the skin. This is supported by the finding of higher levels of antimicrobial peptides, chemokines, and distinct Th17 presence in SGR skin compared to SGP skin [39].

Sebum-derived FFAs enhance innate immune defences by increasing the production of beta-defensin-2 in sebocytes, thus providing antibacterial activity against skin pathogens such as *Cutibacterium acnes* (*C. acnes*) [40]. Furthermore, sebocytes show immune competence, responding to Toll-like receptor (TLR) activation by prioritising pro-inflammatory functions over lipid metabolism [41]. A recent study demonstrated that sebocytes, independently of sebum production, also function as immune modulators, contributing to T cell polarization towards the Th17 phenotype and integrating into the innate immune response [42]. In addition, the holocrine secretion releases the histone H4, a key component of sebocyte antibacterial activity, which enhances the antimicrobial properties of sebum FFAs [43].

The immunological functions of the SGs are regulated by various factors, including sex steroids, neuropeptides, and endocannabinoids, and are implicated in many skin diseases such as acne and atopic dermatitis [13].

### 3.4. SG Role in Crosstalk with Other Cell Populations

Sebocytes are now recognised as key players in the crosstalk between various cell populations within the skin. Numerous studies highlight the critical role of sebocytes in maintaining epidermal integrity and homeostasis through their interactions with other skin cell types. For instance, Nikolakis et al. developed a co-culture model of explant skin with immortalised SZ95 sebocytes, demonstrating that sebocytes enhance the differentiation and proliferation of keratinocytes. These beneficial effects on skin homeostasis are likely mediated by various growth factors and hormones produced by sebocytes, such as sex steroids (e.g., oestrogens and androgens), which are known to promote keratinocyte proliferation and maintain skin tissue homeostasis [44]. Further studies have corroborated this crosstalk, showing that skin cultured without sebocytes exhibits signs of degeneration, underscoring the importance of sebocytes in maintaining skin integrity [45,46]. Additionally, research has demonstrated that factors derived from SZ95 sebocytes influence fibroblast behavior and play a crucial role in preserving epidermal melanocytes, promoting their proliferation, dendricity, and melanogenesis [47,48,49].

**Figure 1:**
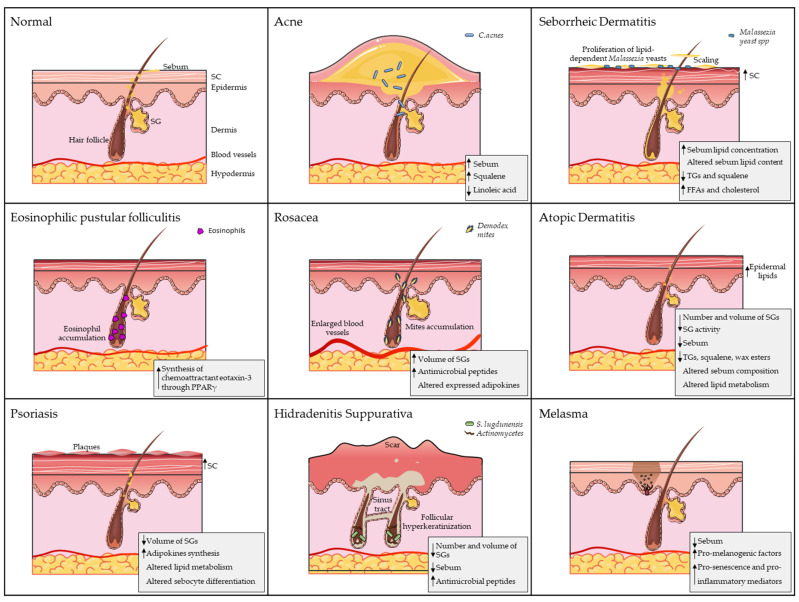
The role of sebaceous glands (SGs) in dermatological diseases. Each panel illustrates a distinct pathology, highlighting disease-specific alterations. Key SG-related pathological features are summarised in the bottom-right corner of each panel. Artworks shown in figure were adapted from pictures provided by Servier Medical Art (Servier; https://smart.servier.com/) (accessed on 10 March 2025), licensed under a Creative Commons Attribution 4.0 Unported License.

Alterations in sebaceous gland function, such as dysregulation of sebum production or composition, can lead to skin disorders or dry skin conditions (Figure 1), highlighting the delicate balance required for healthy skin function.

## 4. Acne

Acne is certainly the most representative pathological condition of the pilosebaceous unit, characterised by blackheads, whiteheads, and pus-filled, sometimes painful, bumps.

It affects up to 80% of adolescents, and according to the latest epidemiological data, its incidence is also increasing in the adult population [50]. The interplay and complex interaction among different factors are responsible for the onset and development of this skin disorder. However, alterations in the function of the SG, leading to the deregulation of its associated downstream product, namely sebum, play a central role in the pathogenesis of the disease. Indeed, sebum overproduction (seborrhoea) combined qualitative sebum changes (dysseborrhoea) can influence other pathogenetic factors traditionally implicated in acne, such as *C. acnes* colonization, follicular keratinization, and inflammation [50,51].

Increased sebum secretion is widely recognised as an important initiating event in acne development and progression, but it is not fully understood whether it is directly related to the severity of the disease [52]. Certainly, sebum overproduction can be associated with *C. acnes* overgrowth. The increased availability of sebum produces an anaerobic and nutrient-rich environment, promoting, in particular, the proliferation of *C. acnes* phylotype I strains, which seem to be more closely associated with acne [53,54,55,56]. Porphyrin production by *C. acnes* can promote keratinocyte inflammation by reactive oxygen species generation [57]. Moreover, *C. acnes* metabolism modifies sebum composition through lipase enzyme activity which hydrolyses sebum triacylglycerols into FFAs, which in turn further stimulate inflammation [58,59]. In particular, palmitic acid (PA) has been demonstrated to induce pro-inflammatory cytokine production in keratinocytes through NF-kB activation [60]. Some experimental evidence also suggests that *C. acnes* can stimulate sebocyte lipid production by releasing soluble factors, leading to a mechanism of exacerbation between sebum production and bacterial colonization [61,62,63].

Changes in the concentration of FFAs can expose SG to stimuli affecting sebum synthesis as well as inflammatory processes. The origin of these fatty acids acting on sebocytes is still unclear, but it could be of double origin: on the one hand, circulating fatty acids from the dietary supply, and on the other hand, the fatty acids directly produced by sebocytes together with those obtained by *C. acnes’* hydrolysis of sebum. In the latter case, we could speak of an autocrine effect: indeed, sebaceous lipids have been shown to penetrate the dermis from the surface or through the infundibulum, reaching various cells, including sebocytes themselves [64]. The different FFAs selectively modulate sebocyte functions. Palmitic acid (PA) emerges as a potent pro-inflammatory agent targeting sebocytes [65]. It acts through the activation of TLR2 and TLR4, stimulating the production of IL-6 and IL-8. It also triggers the NLRP3 inflammasome via the production of ROS, leading to the release of IL-1β. The increase in PA in acne lesions suggests a key role in local inflammation [66,67,68]. Similar to PA, linoleic acid (LA) also exerts an inflammatory effect on sebocytes both directly by stimulating IL-6 secretion from sebocytes and indirectly as the precursor of arachidonic acid (AA). This unsaturated fatty acid stimulates IL-6 and IL-8 secretion from sebocytes and is involved in the production of inflammatory mediators such as leukotrienes and prostaglandins, which are found in increased amounts in acne-affected skin [64,69]. Moreover, AA is involved in phospholipid and neutral lipid formation and thus has a key role in the differentiation and metabolism of sebocytes [70]. Finally, AA participates in the inflammatory reaction through the modulation of the ion channel transient receptor potential vanilloid 3 (TRPV3). The increase in TRPV3 in sebocytes induced by AA (and possibly enhanced by *C. acnes*) amplifies inflammation by upregulating TLR2 and activating NF-κB, thereby creating a vicious circle of inflammation [71]. The imbalance of FFAs and their interaction with sebocytes via specific inflammatory pathways are key mechanisms linking FFAs stimulation to the inflammatory responses and aggravation observed in acne. Moreover, recent findings indicate that FFAs, and in particular AA, can be implicated in the induction of autophagic phenomena [72]. This overstimulation of autophagy could be relevant for a better comprehension of the sebocyte response to lipid stress due to excessive or imbalanced lipid accumulation and thus for acne development and progression.

The altered functionality of the SG is also reflected by compositional changes in the overproduced sebum, and this sebum quality imbalance is considered principally responsible for acne development more than for seborrhoea alone [51]. Decreased levels of essential fatty acids, particularly linoleic acid (LA), are a hallmark of acne sebum [73]. LA deficiency in wax esters, which is associated with hyper-keratosis of the follicular duct and ultimately comedone formation, is probably caused by the dilution effect of increased lipogenesis due to overactive SG. In addition, sebum fatty acids can penetrate the epidermis and then can be incorporated into the epidermal lipids. Thus, the LA depletion observed in acne affects acyl-ceramide composition, contributing to skin barrier impairment and resulting in both increased transepidermal water loss (TEWL) and increased inflammatory mediator permeability of the comedonal wall [59,73,74,75,76]. The increase in the pro-inflammatory lipid fractions, such as mono-unsaturated fatty acids (MUFAs) and lipoperoxides, are other acne-associated sebum features leading to acne lesion development. Sapienic acid (C16:1Δ6), a specific and the most abundant MUFA in sebaceous lipids, produced from palmitic acid (C16:0) by delta-6-desaturase-2 (FADS2) activity, seems to be associated with acne development and severity [11,38,77]. Oxidised lipids, mainly squalene peroxides, are related to comedone formation and inflammatory processes, being able to induce keratinocyte hyperproliferation, lipoxygenase (LOX) activity, and pro-inflammatory cytokine IL-6 release [78,79,80,81,82].

The causes of deregulated SG functionality leading to sebum changes, and the underlying sebocyte defects, are still unclear. The physiological increase in androgens during puberty, which typically coincides with the onset of acne, has long been considered the principal pathophysiological factor of the disease, due to the stimulation of SG growth and activity, and thus sebum production. The correct functionality of the androgen receptor (AR) expressed by sebocytes, as observed in the SEB0662 AR+ cell line, is necessary to achieve complete cellular differentiation, which leads to the accumulation of neutral lipids finally reversed on the skin surface as a holocrine secretion [83]. Otherwise, a marked lack of sebum is observed in subjects with AR gene mutations and androgen-insensitive ones, confirming AR’s role in SG function [84,85]. Over the years, insulin and insulin-like growth factor-1 (IGF-1) have been identified as other important hormonal triggers affecting sebum production. Sebocyte differentiation, in fact, is linked to IGF-1. Higher expression of IGF1R is found in immature sebocytes than in fully differentiated ones [86,87]. The induction of the insulin/IGF-1 axis is also experienced during puberty, and, in addition, IGF-1 is involved in enhancing androgen signalling through different mechanisms, including the stimulation of gonadal and extra-gonadal androgen synthesis; the induction of 5-alpha-reductase activity, with the consequent production of the potent AR ligand dihydrotestosterone; and the inhibition of FoxO1 transcription factor, a nuclear corepressor of AR [88,89,90,91]. Patients affected by acne present higher serum levels and epidermal expression of IGF-1, and subjects with the IGF-1 congenital deficit, such as in Laron syndrome, never develop the disease [92,93,94,95].

The importance of the insulin/IGF-1 pathway in acne pathogenesis is also strengthened by the higher prevalence of acne in Western countries, therefore highlighting the role of the high-glycaemic load diet in acne onset and exacerbation. Hyperinsulinemia and insulin resistance are associated with acne, and post-adolescent acne patients show increased glycemia levels [16,86,96,97]. Otherwise, a diet that reduces the glycaemic load results in insulin sensitivity improvement and in the reduction in sapienic acid, which is correlated with a decrease in acne severity [98,99,100]. Moreover, an in vitro study on SZ95 sebocytes demonstrated that leptin, an adipocyte-secreted hormone in response to increased lipid uptake, increases levels of unsaturated fatty acids, particularly sapienic acid, thus resembling an ‘acne-like’ lipid profile. These findings provide a further link between diet and acne, although additional studies are needed [101]. Lipid synthesis and glucose metabolism are interconnected. De novo lipogenesis (DNL), which is the main metabolic pathway for sebum production within the SG, synthesises fatty acids from glucose. The insulin/IGF-1 pathway over-stimulation in acne patients results in the hyper-stimulation of DNL and thus in excessive and deregulated sebum production [102]. The combination of the effects of androgens and insulin/IGF-1 (which come with pubertal hormonal changes) and the hyperglycaemic diet stimuli results in a synergistic effect as they all converge on the activation of the AKT/mTORC1 pathway. Androgens activate it through the serine/threonine kinase mammalian target of rapamycin complex 2 (mTORC2), while insulin/IGF-1 does so via phosphoinositide 3-kinase (PI3K) signalling [97]. The resulting effect on the modulation of sebogenesis is due to the upregulation of sterol response element binding protein-1 (SREBP-1), a key lipogenic enzyme, resulting from mTORC1 activation and the concomitant reduction in nuclear levels of the transcription factor FoxO1 via the PI3K/AKT pathway. Moreover, the positive activation of HIF-1α through PI3K/AKT signalling produces lipid accumulation together with inflammatory mediator production. Increased levels of perilipin 2 (PLIN2), a protein regulating lipid accumulation in sebocytes, is observed in SZ95 with upregulated HIF-1α, linking seborrhoea and inflammation [68,103]. Activation of the AKT/mTORC1 pathway is therefore now recognised as crucial in the onset and development of acne. A variety of new pharmacological agents are being tested for their capacity to manage the stimulation of this pathway, which leads to lipogenesis and inflammation [104,105,106,107,108].

The sebocyte differentiation process is fundamental for lipid synthesis and the incomplete differentiation of sebocytes is probably associated with a greater susceptibility to insulin/IGF-1 stimulation as well as to other hormonal stimuli, such as androgens. The altered response, rather than the excessive stimulus, may therefore be responsible for the deregulated functionality of the SG. Poorly differentiated SZ95 sebocytes express higher levels of insulin and IGF-1 receptors, together with the upregulation of AKT/mTOR signalling, resulting in higher sensitivity to hormone stimuli in comparison with more differentiated cells [109]. Moreover, a low differentiation grade of SZ95 sebocytes is also associated with lower PPARγ levels, in accordance with the correlation found between PPARγ expression and sebocyte differentiation in the peripheral and central zone of the SG [110]. These findings regarding increased AKT/mTOR signalling together with the reduced expression of PPARγ resemble what is observed in the skin of acne patients. Moreover, insulin-induced lipogenesis produces in SZ95 sebocytes alterations that mimic acne sebum in terms of lipid composition and inflammatory mediators [38,59,70,94,109,111]. The insulin/IGF-1 stimulation can be counteracted by PPARγ induction through a selective modulator, allowing sebocytes to reach a differentiation grade suitable for the correct response to insulin/IGF-1 stimulation [109]. The importance of the maturation process in sebocytes for their correct functional mechanisms has been highlighted by recent findings indicating that different stages of SZ95 maturation are associated with different susceptibility degrees to doxycycline treatment [112]. Acting on sebocyte differentiation is thus considered a new therapeutic strategy for acne treatment that is able to rebalance disrupted sebum compositional changes [109,113].

## 5. Seborrheic Dermatitis

Seborrheic dermatitis (SD) is a chronic, relapsing, inflammatory skin condition characterised by erythematous, yellow or white, greasy, scaly plaques with variable presentation depending on skin phototype, age, and anatomical location. SD affects approximately 1% to 3% of immunocompetent adults and is more common in men. It typically localises to areas of the body rich in SGs, such as the scalp, face (nasolabial folds, eyebrows, ears) and upper trunk, and has a significant negative impact on patients’ quality of life in the form of psychological distress or low self-esteem. SD has a multifactorial pathophysiology and at least three factors have been proposed as essential for its development: skin surface lipid secretion by SGs, colonization by commensal *Malassezia yeast* spp., and individual susceptibility in terms of immunological dysregulation that predisposes the patient to SD [114,115,116,117,118,119]. According to the proposed pathophysiology, SD is caused by the stepwise succession of the following events: abnormally increased and altered sebum lipid concentration by SGs, which creates a rich environment for the proliferation of lipid-dependent microorganisms; *Malassezia* yeast therefore flourishes, secreting lipases and phospholipases that hydrolyse sebum triglycerides into free fatty acids and lipid peroxides, causing further *Malassezia* growth and inflammatory response. The immune system triggers cytokine release from Th2 and Th17 cells, neutrophils, and dendritic cells, as well as complement activation. These alterations lead to keratinocyte proliferation and stratum corneum thickening, increased inflammation and ultimately skin barrier disruption and ceramide loss, resulting in clinically evident erythema, pruritus, and scaling [116,117,120,121,122].

The role of sebaceous activity in the etiology of SD is suggested by its temporal predilection during infancy (cradle cap), puberty, when SGs are turned on under sex hormone control, and adulthood (affecting those 30–60 years of age) in areas of high sebaceous activity [115,123,124]. The role of specific sebum fatty acids becomes important in the etiology of SD when *Malassezia* metabolism is considered. The lipophilic yeast does indeed degrade sebum, releasing several fatty acids from triglycerides. However, it consumes the very specific saturated fatty acids required for its proliferation, leaving the irritant unsaturated fatty acids, such as oleic acid and arachidonic acid, on the skin, causing the subsequent inflammatory response [121,123,125]. Experimentally, human primary sebocytes exposed to different *Malassezia* strains in a co-culture system showed a trend towards increased sebum lipid production [126]. Furthermore, the lipid profile analysed in sebum collected from SD patients revealed changes in sebum composition over time as a direct result of *Malassezia* metabolism. Indeed, after treatment with an antimicrobial shampoo that removes scalp microflora, sebum composition returns to near normal levels of triglycerides and free fatty acids [123]. However, some clinical evidence indicates that SD patients may have normal sebum production and that individuals with excessive sebum production do not necessarily develop SD. These data suggest that although SG activity is strongly correlated with SD, sebum production alone is not its determinant [115]. In addition to levels of sebum production, alterations in lipid composition may also play a role in the development of SD, potentially creating a favourable environment for *Malassezia* growth. In patients with SD, triglycerides and squalene are reduced, while free fatty acids and cholesterol are significantly increased as a result of triglyceride breakdown by *Malassezia* lipases [123]. Some evidence suggests that an individual’s sensitivity to irritating unsaturated fatty acids, such as oleic acid, and the resulting inflammatory response, ultimately contribute to their susceptibility to developing SD. When oleic acid is applied topically, patients with SD exacerbate skin desquamation, whereas unaffected subjects have negligible effects, demonstrating their underlying increased sensitivity to fatty acid-induced skin barrier disruption [121].

SD has an increased prevalence in many neurological and psychiatric disorders [127]. Approximately 52–59% of patients with Parkinson’s disease (PD) are affected by SD [128]. The pathophysiology of this association is complex and includes facial immobility, which contributes to enhanced sebum accumulation, and an increase in plasma levels of α-melanocyte stimulating hormone (α-MSH), a neuropeptide responsible for increased sebum production by SGs [129]. PD patients also have a deficit in α-MSH inhibition due to reduced dopamine levels. Therefore, PD patients are likely to have elevated sebum levels and develop DS. In addition, antiparkinsonian drugs such as L-dopa are effective in reducing sebum secretion and treating the SD found in these patients [115,128].

## 6. Eosinophilic Pustular Folliculitis

The sebaceous gland is increasingly recognised as a key player in the pathogenesis of eosinophilic pustular folliculitis (EPF), a rare inflammatory skin disorder characterised by recurrent pruritic papules, pustules, and eosinophil-rich infiltrates [13,130]. EPF is mainly seen in Japan and China, but cases have also been reported in Europe and North America [131], with a widely variable prevalence.

Histologically, EPF is characterised by eosinophilic infiltration of hair follicles and sebaceous glands [132]. Data in the literature indicate that a T-helper 2 (Th2) cytokine-dominant condition (e.g., IL-4, IL-5, and IL-13) is a hallmark of EPF [133,134]. Furthermore, Sato et al. identified the upregulation of IL-36 cytokines in EPF, which are chemoattractive for neutrophils and eosinophils [135].

The pathogenesis of EPF is thought to be mediated by prostaglandin (PG)D2, which induces the production of the chemoattractant eotaxin-3 in sebocytes via PPARγ activation, potentially explaining the eosinophilic infiltration around pilosebaceous units [13,136]. This mechanism is supported by the efficacy of the EPF treatment with indomethacin, an inhibitor of the enzyme cyclooxygenase (COX), which is central to the generation of prostanoids such as PGD2 [136,137].

Thus, the pathophysiology of EPF is not yet fully understood, but the sebaceous gland may play a critical role in the process leading to eosinophil accumulation. These findings are supported by the clinical observation that EPF often occurs in sebaceous-rich areas, such as the face and scalp [13,131].

## 7. Rosacea

Rosacea is a chronic inflammatory skin condition that primarily affects the face and is characterised by recurrent flushing, erythema (redness), telangiectasia (visible blood vessels), papules, pustules, and sometimes ocular lesions and rhinophyma. Based on these symptoms, rosacea is commonly subdivided into four subtypes: ocular rosacea (OR), papulopustular rosacea (PPR), phymatous rosacea (PhR), and erythematotelangiectatic rosacea (ETR). Rosacea affects approximately 10% of the global population [138,139,140].

Rosacea predominantly affects sebaceous gland-rich facial areas [141,142]. A specific rosacea subtype, PhR, has also been associated with skin thickness due to SG hyperplasia [141,142,143]. These findings suggest that SGs may play a crucial role in the pathogenesis of the condition. Studies employing topical isotretinoin (13-cis-retinoic acid) have provided evidence supporting this hypothesis, demonstrating a significant decrease in sebaceous gland volume and sebum production, as well as an improvement in the erythema and papulopustules of rosacea patients [142,144,145,146].

The precise etiology of rosacea has yet to be fully elucidated, although it is hypothesised to be multifactorial, involving a combination of genetic, environmental, vascular, and immunological factors [140,147,148]. Patients with rosacea are characterised by an altered skin microbiome, with a significantly higher population of microorganisms such as *Demodex* mites, which normally reside in the pilo-sebaceous follicle [149,150]. *Demodex* mites may modulate sebocytes immune reactions, potentially contributing to the disorder’s pathogenesis. Lacey et al. demonstrate how *Demodex* mites are able to influence the expression of important pro-inflammatory mediators (e.g., TNFα) in SZ95 sebocytes, with a tendency towards an initial downregulation followed by a subsequent upregulation, particularly when the number of mites is high. In response to the *Demodex* challenge, the researchers also observe an increase in TLR2 expression, although this increase did not reach statistical significance [151]. However, an increased expression of TLR2, with other key features like high levels of pro-inflammatory antimicrobial cathelicidin peptides, characterise the immune response in rosacea epidermis [152,153]. Data in the literature indicate that SZ95 sebocytes can also increase the expression of cathelicidin peptides, like keratinocytes, following exposure to stimuli such as vitamin D3 or *C. acnes* [154]. Therefore, sebocytes could participate in the pathogenesis of rosacea by promoting an inflammatory state of the skin, as is seen with keratinocytes. The exact sequence of events has yet to be established.

Data in the literature explore other possible mechanisms by which SGs may contribute to the pathogenesis of rosacea. Kovács et al. identify the altered presence of several adipokines, such as SERPINE1, in SGs from rosacea biopsies, highlighting the crucial role of sebocytes in maintaining the integrity and health of the skin barrier [155,156]. Furthermore, the involvement of sebaceous glands in rosacea is further supported by the identification of serum amyloid A (SAA) as a marker of activated sebocytes in rosacea skin samples, triggering IL-1β activation and secretion [41]. Moreover, Dajnoki et al. demonstrated a higher expression of thymic stromal lymphopoietin (TSLP), an immunomodulatory cytokine, in rosacea sebaceous gland-rich skin compared to sebaceous gland-poor skin. Notably, linoleic acid, a major component of sebum, can induce TSLP expression in keratinocytes [157]. This suggests that altered sebum composition and sebocyte activity may contribute to the inflammatory milieu observed in rosacea, but further research is needed to fully clarify the pathogenesis of the condition.

## 8. Atopic Dermatitis

Atopic dermatitis (AD) is a chronic, relapsing, intensely pruritic, inflammatory skin disease with a complex pathophysiology, characterised by a broad spectrum of clinical manifestations such as dryness, redness, and severe itch. It is often associated with other atopic manifestations such as food allergies, allergic rhinitis, and asthma [158]. AD affects 15–20% of children and 1–3% of adults worldwide [159,160,161]. The disease is associated with skin barrier dysfunction characterised by increased transepidermal water loss (TEWL) and decreased stratum corneum (SC) hydration, leading to reduced protection against microbial infections, irritants, allergens, and environmental factors [162,163,164]. A major hallmark of AD is the increased production of IL-4 and IL-13, both type 2 cytokines that drive the inflammatory process in AD [165,166,167,168].

Although most studies focus on the dysfunctional synthesis of barrier-related proteins and skin surface lipids, especially ceramides, in keratinocytes [163,169,170], a link between altered SG function and skin barrier dysfunction appears to contribute to the development of the disease [13,117,171], also considering that sebum lipids amounts, due to different SG density, can influence SC lipid metabolism and shape the expression of epidermal permeability barrier lipids [2]. Wirth and co-authors [172] demonstrate a significant reduction in the number/skin area and volume of SGs in the unaffected skin of AD patients, accompanied by a prolonged S-phase in sebocytes, indicating a slower rate of cell proliferation and reduced SG activity. Rajka [173] found a reduction in SG lipids, mainly squalene and wax esters, with a corresponding increase in epidermal lipids, mainly cholesterol, on the back of the hands of AD patients, consistent with clinical observations of frequent dryness of the skin in this region. Further studies show that the amount of sebum secreted by SGs is reduced in the clinically unaffected skin of AD patients and is negatively correlated with barrier function, skin hydration, and disease severity [174,175,176,177,178]. In support of this, patients with acne, a condition associated with increased sebum production, have a reduced incidence of AD compared to healthy subjects [179], and AD patients had a reduced risk of being treated for severe acne [180]. Lipidomic analysis of sebum from the non-lesional skin of AD patients and healthy controls also reveals changes in sebum composition. Among the differential lipid subclasses, triglycerides were exclusively downregulated in AD patients and correlated with disease severity [178]. Another skin lipidomic study showed that squalene was significantly depleted in AD skin and that the effect of AD disease was exacerbated in males, resulting in more severe lipid depletion [181]. Furthermore, transcriptomic data reveal alterations in the expression of lipid metabolism-related genes associated with skin inflammation and barrier dysfunction in a combined cell population derived from inner root sheath keratinocytes and SG cells in non-lesional AD skin [178,182]. In support of these data, spatial transcriptomics of SG in AD skin samples shows alterations in genes mainly related to lipid metabolism, in particular cholesterol biosynthesis, fatty acid metabolism, and steroid metabolism, and inflammation [183]. Importantly, Zhang and co-authors [184] linked the cytokine milieu of AD to sebocyte function by showing that IL-4 and IL-13 upregulate the expression of 3β-hydroxysteroid dehydrogenase 1 (3β-HSD1), a key rate-limiting enzyme in the biosynthesis of sex steroid hormones from adrenal precursors, which is predominantly expressed by SGs in human skin [185]. The same study shows that IL-4 and IL-13 induce 3β-HSD1 expression through the activation of STAT6, promote 3β-HSD1-dependent androgen synthesis, and induce lipid abnormalities in human sebocytes, in particular a decrease in the total amount of triglycerides. Consistent with these in vitro findings, 3β-HSD1 expression is elevated in the skin of AD patients and can be restored by treatment with the IL-4Rα monoclonal antibody, dupilumab [183,184]. These results suggest that type 2 cytokines may alter skin lipid production in AD through the regulation of sex steroid hormone synthesis in the SG [184]. Of particular interest is TSLP, a crucial cytokine in promoting type 2 immune responses that is highly expressed in AD lesions and contributes to disease severity and persistence. A recent study demonstrates the ability of TSLP to modulate sebum secretion and SG maturation/turnover, not by directly affecting SG activity, but through IL-4/IL-13 induction from Th2 cells [186,187]. Moreover, Lin and co-authors [188] found that SGs and sebocytes expressed IL-4 receptors and produced high levels of Th2-associated inflammatory mediators after IL-4 exposure. Among these, galectin-12 is a lipogenic factor affecting sebocyte differentiation/proliferation that promotes CCL26 expression in a PPARγ-dependent manner, inducing an inflammatory response around the SGs and an AD-like phenotype in a mouse model [188].

Supporting the concept that SGs are not bystanders in inflammatory skin diseases but can actively and differentially modulate inflammation and the commensal bacteria balance, a study reported that the levels of sebum and its microbial metabolite propionic acid are lower on the skin surface of AD patients compared to healthy controls. In addition, topical propionate attenuates skin inflammation in the MC903-driven mouse model of AD by counteracting IL-33 production in keratinocytes through the inhibition of histone deacetylase and regulation of the aryl hydrocarbon receptor signalling pathway [177]. The cytokine IL-33 is often considered an upstream inducer of the type 2 immune response, and its expression is significantly increased in keratinocytes of lesional skin from AD patients [189,190]. Finally, topical propionate improved skin symptoms in AD patients in a proof-of-concept clinical trial [177].

## 9. Psoriasis

Psoriasis (PSO) is a chronic, immune-mediated inflammatory skin disorder characterised by the rapid proliferation and altered differentiation of keratinocytes, which contribute to the formation of a thickened epidermis [191,192]. Psoriasis can show up as a variety of clinical cutaneous symptoms, but the most common ones are symmetrical papules and plaques that are persistent [193]. Furthermore, patients with PSO are more likely to develop other serious and chronic conditions: psoriatic arthritis, metabolic syndrome or its components, cardiovascular problems, depression, non-alcoholic fatty liver disease, Crohn’s disease, and lymphoma are examples of these comorbidities. PSO affects 2–3% of the total population [191,192].

The pathogenesis of PSO involves a complex interplay between genetic predisposition, environmental triggers, and immune dysregulation, with the involvement of immune mediators, such as IL-23 and IL-17, which drive the inflammatory cascade [194,195]. Recent data in the literature have shown an increased interest in the role of the SG in the pathogenesis of PSO, as the morphology of the SG appears to be altered in psoriatic skin compared to normal or non-lesional skin. Liakou et al. measured a gland volume of 0.018 mm^3^ in psoriatic lesions versus 0.057 mm^3^ in non-lesional skin, i.e., a significant reduction of 68% [196]. Additionally, Rittié et al. reported a 91% average reduction in gland volume in psoriatic areas outside the scalp [197]. These findings indicate that, in psoriasis, SG characteristics differ markedly from those of non-lesional or healthy skin. In addition to these observational studies, the biological role of sebocytes was therefore investigated. Sebocytes in psoriatic lesions show altered differentiation and activate inflammatory pathways [196]. Data in the literature show that sebocytes both express and secrete various adipokines such as IL-6, visfatin, adiponectin, and leptin, which are involved in the pathophysiology of PSO. Visfatin specifically demonstrated the ability to enhance the development of psoriasis by stimulating human keratinocytes to produce increased levels of antimicrobial peptides [155,156,198]. Furthermore, sebocytes express several genes associated with inflammation in PSO, including SERPINE1. They have also been shown to be associated with alterations in other psoriasis-specific pathways through the expression of genes related to lipid metabolism (e.g., *ACOT4*), keratinization (e.g., *KRT5/7/16*), neutrophil degranulation, and antimicrobial peptides (e.g., *S100A7-9*) [155,156,183].

In conclusion, further studies are needed to better elucidate whether sebocytes play an active role in initiating the pathogenesis of psoriasis or whether they are subject to the effects of psoriatic changes that have already been initiated, as this could significantly advance our understanding of the disease and lead to more targeted therapeutic strategies.

## 10. Hidradenitis Suppurativa

Hidradenitis suppurativa/acne inversa (HS) is a chronic inflammatory skin disease of the hair follicle that usually presents after puberty with painful deep abscesses and inflamed lesions in the apocrine gland-rich areas of the body, such as the axillae, groin, mammary, and anogenital regions, with a high physical and psychological impact on patients. It affects about 1% of the global population, with a female prevalence. HS is a multifactorial disease whose pathogenesis is not fully understood, with genetic and lifestyle factors such as smoking and obesity playing a key role. Typical HS lesions are characterised by suppuration, extensive sinus tracts, scarring, and keratinised tunnels that elongate and branch in the dermis as the disease progresses, trapping bacteria, with *Staphylococcus lugdunensis* and anaerobic *actinomycetes* being the most prevalent species [199], which exacerbate local chronic inflammation [200,201]. The key pathogenetic event in HS is considered to be the occlusion of terminal hair follicles, caused by hyperkeratinization and hyperplasia of the follicular epithelium, followed by cyst development and rupture, causing perifollicular lympho-histiocytic inflammation, ultimately leading to the destruction of the pilosebaceous unit [202]. Current evidence suggests that HS is an autoinflammatory skin disease associated with alterations in the innate immune system [203,204]. Elevated levels of several pro-inflammatory cytokines, most notably TNF-α, IL-1β, IL-17, and IFN-γ, have been observed in HS lesions, together with dysregulated function of other immune players closely linked to macrophage function, including the overexpression of matrix metalloproteases (MMPs), the upregulation of toll-like receptors, the upregulation of the (NOD)-like receptor protein 3 (NLRP3) inflammasome, dysregulated keratinocyte function, and impaired Notch signalling [200,201,204,205,206], which plays a crucial role in the differentiation of hair follicle and maintenance of skin appendages, including SGs [203]. In Notch-deficient mice, SGs failed to form [207]. In hair follicles from the perilesional skin of patients with HS, the number and volume of SGs were reduced [208]. The metalloproteinase ADAM10 is required for the proteolytic cleavage of the extracellular domain of the Notch receptor, a step which is necessary for its signalling activity. It has been shown that impaired Notch signalling in the mouse epidermis due to disruption of ADAM10 leads to hair loss, hyperplasia, epidermal cyst formation, the loss of SGs and impaired skin barrier function [209]. Interestingly, all the major histopathological abnormalities in ADAM10- or Notch-deficient mice mimicked the histopathological alterations found in HS. Given the central role of the SG in the local endocrine homeostasis of the skin, it can be hypothesised that some of the features of HS may indeed be a consequence of the absence of individual functions of the SGs, such as the synthesis of hormones, antibacterial lipids and peptides, and pro-inflammatory chemokines and cytokines. Furthermore, reduced amounts or the absence of sebum may lead to increased friction in the infundibulum of the hair follicle, resulting in follicular hyperkeratosis in the initiating steps of HS pathogenesis [208].

A higher prevalence of diabetes mellitus has been observed in patients with HS compared to controls [210,211,212]. Insulin and insulin-like growth factor 1 (IGF-1) lead to the activation of mammalian target of rapamycin (mTOR), whose expression was found to be increased in both the lesional and non-lesional skin of HS patients compared to the normal skin of healthy controls, with a strong correlation to disease severity [94,213,214]. Overexpression of mTOR in HS lesions further supports the production of IFN-γ and IL-17 [201]. Interestingly, mTOR signalling is involved in promoting adipogenesis and lipogenesis, as well as the accumulation of triacylglycerols in SGs [94,215]. Notably, the model currently considered optimal for ex vivo studies of HS is the 3D-SeboSkin model, in which explants from the lesional and perilesional skin of HS patients are co-cultured in direct contact with SZ95 sebocytes as a feeder layer. This model resulted in the overall improved structural integrity of both lesional and perilesional skin, the preservation of the proliferating epidermal basal layer, and the maintenance of the differential expression and pattern of HS skin markers S100A9, KRT16, and SERPINB3 in the epidermis, dermis, and appendages of HS lesional and perilesional skin compared to healthy skin, suggesting that sebocytes provide essential factors to preserve the vitality of skin explants [45,46,216]. Zouboulis and co-authors [216] performed whole genome profiling of lesional, non-lesional, and healthy control skin to identify dysregulated genes associated with HS pathology. Sixteen genes were identified to characterise HS from a molecular point of view. Subsequent immunohistochemical analysis of the corresponding protein expression in skin compartments revealed that, among the proteins highly expressed in HS lesions, the member of the antimicrobial protein family produced by SGs, small prolin-rich protein-3 (SPRR3) [217], was detected in apocrine sweat gland ducts and SGs, suggesting the involvement of skin glands in HS.

## 11. Melasma

Melasma is an acquired hyperpigmentary disorder that manifests primarily as asymptomatic light to dark brown patches symmetrically distributed on the photo-exposed areas of the face, causing significant cosmetic and psychological distress to patients [218,219]. It typically affects women and occurs more frequently in individuals with intermediate Fitzpatrick phototypes III–V and rarely in extreme skin types. It is a multifactorial disease involving a predisposing genetic background, hormonal factors, and exposure to solar radiation. Melasma has features of photoaging [218,220,221,222], with solar elastosis, skin barrier dysfunction, basement membrane modification, chronic inflammation, increased dermal mast cells and vascularity, and impairment of the intercellular skin network with the overexpression of keratinocyte- and fibroblast-derived paracrine melanogenic factors [223,224,225,226,227,228,229,230,231,232,233]. Although the face is widely exposed to sunlight, melasma manifests mainly in specific regions where sebaceous glands (SGs) are abundant, such as the malar area, forehead and upper lips. Some data in the literature suggest that sebocytes may exert paracrine effects on melanocyte function and may contribute to the distribution of melasma lesions on the face. Areas of the body rich in sebocytes, such as the face, axillae, and genitalia, are hyperpigmented and have the highest melanocyte density [234]. When co-cultured with the human sebaceous cell line SZ95, human epidermal melanocytes increase dendrite formation and proliferation [47]. Furthermore, preservation of epidermal melanocyte integrity has been observed in an ex vivo co-culture skin model with SZ95 sebocytes (3D-SeboSkin) [48]. SGs secrete several cytokines and growth factors, including IL1α, IL6, angiopoietin, and adipokine, which can modulate melanocyte function [155,221]. In addition, UV-oxidised skin surface lipids, particularly squalene derived from SG activity, induce melanocyte proliferation and melanin synthesis in organ tissue cultures, acting as mediators capable of mediating epidermal cell changes [235]. A reduced rate of facial sebum excretion has been observed in patients with melasma, suggesting the role of SG regulation in the development of the disease [236].

Recently, the involvement of SGs in the physiopathology of melasma has been demonstrated [49]. In particular, following UVA irradiation, SZ95 sebocytes, like keratinocytes, are able to produce and upregulate pro-melanogenic factors such as α-MSH, EDN1, and the stromal sustaining factors SCF and b-FGF. They also produce and upregulate pro-inflammatory cytokines and lipid-derived mediators, such as arachidonic acid, LTB4, and prostaglandins, which are involved in inflammation, pigmentation, and the aging process as senescence-associated secretory phenotype (SASP) factors [237,238,239]. Irradiated SZ95 sebocyte-conditioned media induced pigmentation in melanocytes and the expression of senescence markers, pro-inflammatory cytokines, and melanogenic growth factors in fibroblasts. Furthermore, the pigmentation produced in human skin explants maintained in co-culture with SZ95 sebocytes confirmed the influential role of sebocytes in promoting melanogenesis, thus reinforcing the concept that increased melanocyte activity in melasma is associated with a local inflammaging process involving the dermal compartment in which sebocytes play a key role.

## 12. Conclusions

A considerable amount of research has shown that the SG is a very active organ, contributing significantly to numerous homeostatic functions essential for skin health and integrity. In addition to its important role in the developmental biology of the pilosebaceous unit, the SG is involved in the maintenance of skin barrier function, local neuroendocrine function, the innate immune response, and the regulation of skin bacterial colonization. Structural and functional disorders of the SG may lead to a perturbation of skin homeostasis and thus contribute to the development of several inflammatory skin diseases (Table 1). A detailed investigation of SG activity and its contribution to skin barrier function is needed and may reveal new therapeutic strategies for the treatment of inflammatory dermatoses.

## Figures and Tables

**Table 1:** Pathological skin conditions and salient SG-related features, along with corresponding references.

Skin Condition	SG-Related Pathological Features	References
Acne	-sebum overproduction -*C. acnes* overgrowth -qualitative sebum changes (linoleic acid decrease, squalene, squalene peroxides, and sapienic acid increase) -inflammatory process -insulin/IGF-1 axis involvement	[38,50,51,52,53,54,55,56,59,77,78,79,80,81,82,86,94,109]
Seborrheic dermatitis	-sebum overproduction -*Malassezia yeast* spp. overgrowth -qualitative sebum changes (oleic acid and arachidonic acid surplus) -inflammatory process	[114,115,116,117,118,119,120,121,123,125,126]
Eosinophilic pustular folliculitis	-eosinophilic chemoattractant eotaxin-3 increase -PPARγ involvement	[133,134,135,136,137]
Rosacea	-SG hyperplasia -pro-inflammatory mediators upregulation (cathelicidin peptides) -adipokine altered presence	[149,150,151,152,153,154,155,156,157]
Atopic Dermatitis	-SG number and volume reduction -SG reduced activity -sebum reduced production -qualitative sebum changes (triglycerides and squalene decrease) -alteration of lipid metabolism-related gene expression -inflammatory process -3β-HSD1 involvement	[172,173,174,175,176,177,178,181,182,183,184,185,186,187,188]
Psoriasis	-SG volume reduction -sebocyte altered differentiation -inflammatory process -adipokine production	[183,194,195,196,197,198]
Hidradenitis suppurativa	-SG number and volume reduction -sebum reduced production -antimicrobial peptides overproduction -Notch signalling impairment	[203,207,208,209,217]
Melasma	-sebum reduced production -production of pro-melanogenic factors -production of pro-inflammatory and SASP factors	[47,48,49,155,235,236]

## Data Availability

No new data were created or analysed in this study.

## Abbreviations

AD: atopic dermatitis; ACOT4: Acyl-CoA Thioesterase 4; ADAM10: ADAM Metallopeptidase Domain 10; AKT: protein kinase B; AR: androgen receptor; C16:1Δ6: sapienic acid; CCL26: C-C Motif Chemokine Ligand 26; COX: cyclooxygenase; CRH: corticotrophin-releasing hormone receptor; DHT: dihydrotestosterone; DNL: de novo lipogenesis; EGF: epidermal growth factor; EDN1: endothelin-1; EPF: eosinophilic pustular folliculitis; ETR: erythematotelangiec-tatic rosacea; FADS2: fatty acid desaturase 2; FFAs: free fatty acids; b-FGF: fibroblast growth factor 2; FoxO1: Forkhead Box O1; HIF-1a: Hypoxia Inducible Factor 1 Subunit Alpha; HS: Hidradenitis suppurativa; 3β-HSD1: 3β-hydroxysteroid dehydrogenase 1; IFN-γ: interferon- γ; IGF-1: insulin-like growth factor-1; IL: interleukin; KRT: keratin; LA: linoleic acid; LOX: lipoxygenase; LTB4: leukotriene B4; MC1-5R: melanocortin receptors; MMPs: matrix metalloproteases; α-MSH: α-melanocyte stimulating hormone; MUFAs: mono-unsaturated fatty acids; NF-kB: nuclear factor kappa-light-chain-enhancer of activated B cells; NLRP3: (NOD)-like receptor protein 3; Notch: Notch Receptor 1; OR: ocular rosacea; OLR1: Oxidised Low Density Lipoprotein Receptor 1; PA: palmitic acid; PD: Parkinson’s disease; PG: prostaglandin; PhR: phymatous rosacea; PLIN2: perilipin 2; PI3K: phosphoinositide 3-kinase; PPAR: peroxisome proliferator-activated receptors; PPR: papulopustular rosacea; PSO: psoriasis; S100A9: S100 calcium-binding protein A9; SAA: serum amyloid A; SASP: senescence-associated secretory phenotype; SC: stratum corneum; SCF: stem cell factor; SD: seborrheic dermatitis; SERPINB3: Serpin Family B Member 3; SERPINE1: Serpin Family E Member 1; SG: sebaceous gland; SPRR3: small prolin-rich protein-3; SREBP-1: sterol response element binding protein-1; STAT6: Signal Transducer And Activator Of Transcription 6; TEWL: transepidermal water loss; TGF-β: transforming growth factor-β; TLR: Toll-like receptor; mTOR: serine/threonine kinase mammalian target of rapamycin complex; TNF-α: tumour necrosis factor- α; TSLP: thymic stromal lymphopoietin.
